# Dimensions of Proximity: An Actionable Framework to Better Understand Integrated Practices in Cancer Networks

**DOI:** 10.5334/ijic.6434

**Published:** 2022-08-16

**Authors:** Dominique Tremblay, Nassera Touati, Susan Elizabeth Usher, Johanne Cournoyer

**Affiliations:** 1 Université de Sherbrooke, Canada; 2 École nationale d’administration publique, Canada; 3 École nationale d’administration publique and Centre de recherche Charles-Le Moyne, Canada; 4 Centre de recherche Charles-Le Moyne, Canada

**Keywords:** proximity, healthcare network, integration, oncology, interpretive description

## Abstract

**Introduction:**

This study empirically explores how dimensions of proximity that support integrated care emerge from deliberate actions within a cancer network in Quebec (Canada).

**Methods:**

We conduct a supplementary analysis of qualitative data from a primary multi-case study focused on collaborative governance and cancer care integration. Data from semi-structured interviews, documents and observation are analysed to find out how relationships take shape through actions that create different dimensions of proximity, and how these contribute to integrated practices.

**Results:**

Deliberate actions at different levels within the network create dimensions of proximity. The creation of committees and communities of practice at national and local level establish geographic proximity. Relational proximity among actors emerges to different degrees in these venues. Cognitive proximity is generated by consistent promotion of the national cancer plan and person-centred care. The priority of cancer care at policy level and prescription of common standards enhance organizational proximity. Synergy between dimensions of proximity appears essential to the emergence of integrated practices. Insufficient efforts to create technological and institutional proximity contribute to inconsistent clinical and professional integration.

**Conclusion:**

The concept of proximity appears a promising complement to existing models of integration, especially in complex contexts such as cancer networks.

**Highlights:**

- Deliberate actions at different levels within the cancer network create a number of dimensions of proximity
- Geographic proximity, be it objective or subjective, facilitates relational, cognitive and institutional proximity
- A national cancer plan sustained by shared leadership enhances organizational proximity, facilitating integrated practices
- Activation of different dimensions of proximity among network actors likely underpins and sustains functional, normative and organizational integration
- Insufficient efforts to create technological and institutional proximity contribute to inconsistent clinical and professional integration

The multiple dimensions of proximity appear a promising complement to existing models of integration, especially in complex contexts such as cancer networks.

## Introduction

Integrated network-based practices in cancer care seek to coordinate inputs across the continuum, which can unfold over a long time-frame, involve multiple specialized and primary care providers, and impact all dimensions of a person’s life [1]. In theory, integration of multiple interconnected levels of healthcare networks contributes to system attributes such as coordination, collaboration, communication, coherence, cost-effectiveness and respect for patient preferences [2,3]. Despite efforts at political, managerial and clinical level, cancer care remains fragmented, with impacts on safety and quality [4].

### Integration in HEALTH CARE as a solution to fragmentation

Valentijn, looking at primary care, conceptualizes a multilevel model, where normative and functional integration mechanisms enable clinical, professional and organizational integration [5,6]. This work makes a significant contribution to the conceptualisation of integrated care, however further work is needed to understand how integrated care mechanisms are activated in specialized domains such as cancer care. In a comprehensive review of studies on the design and implementation of integrated practices in health care, Gonzalez-Ortiz concludes that recommendations are mostly “lists of key building blocks”, rather than “frameworks supporting the process of implementation” [2]. The review identifies 175 elements associated with successful care integration (e.g. structural supports and resource allocation, co-location of services, teamwork and care coordination, shared decision making and problem solving, inter-organizational and inter inter-professional governance, patient-centeredness) [2]. Most of the endogenous elements of integrated care are dependent on context, which changes over time, suggesting dynamic relationships rather than a stable set of building blocks. However, the question of how integrated practices take shape through deliberate actions in shifting contexts has not yet been well addressed, despite its importance to understanding the emergence and persistence of integrated network-based practices. This article focuses on the Quebec Cancer Network and looks to the concept of proximity as a means of understanding how actions taken in the network contribute to integrated practices.

### A case of deliberate actions to integrate cancer care

The Quebec Cancer Network (QCN) represents a typical case to better understand deliberate actions likely to support integrated network-based practices in a specialized domain and changing context [7].

**Figure 1:**
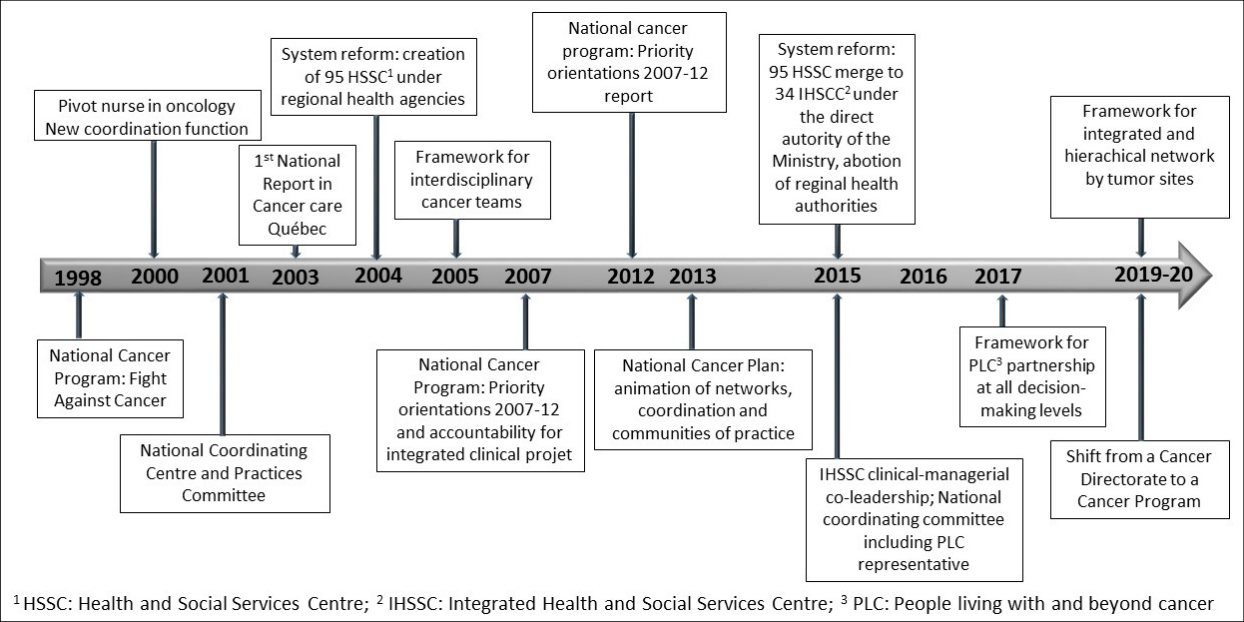
The National Cancer Network in Quebec 1998–2020.

The Ministry of Health and Social Services (MSSS) in Quebec launched its National Cancer Plan in 1998 [8]. Key elements persisted through various iterations of the program: a network model; a whole-person approach covering a range of needs over the entire cancer continuum; inclusion of patient perspectives in decisions; interdisciplinary teamwork; oncology pivot nurses within teams [9]; and a hierarchical organisation of services. Figure 1 highlights the evolution of the national cancer plan through various deliberate actions. Under the central authority of a Cancer Directorate (Quebec Cancer Program since 2019) at Ministry level, coordinating committees were established at national and regional level, professional communities of practice were supported and delivery organizations developed integrated clinical projects. Mandated efforts proceeded from creating a coordination function (pivot nurse) in 2001 within interdisciplinary teams [10,11], to “unified governance of an integrated hierarchical service trajectory” in 2007, to conceptions of “network” and even “network-of-networks” [12]. These efforts evolved within the shifting landscape of the broader health and social service system. Major health system reforms in 2015 redefined territorial boundaries and governance relations between the Ministry, cancer program and leaders of the new Integrated Health and Social Service Centres (IHSSCs), redrawing cancer care teams and referral patterns. Studies of the evolving cancer network considered that turbulence in the healthcare context destabilized efforts at integration [13]. This pointed to a need to identify durable mechanisms that could support and sustain integration across levels.

The “network-of-networks” was seen as an integrative tool in which synergistic components produce new relationships and interactions driving integrated practices. A primary study for which DT was the PI aimed to investigate how, why, by whom, for whom, and under what conditions the collaborative governance attempted in the “network-of-networks” contributes to network-based practices [7]. This primary study consisted of a mixed-methods longitudinal case study in the realist evaluation tradition, with multiple nested cases. Analysis of the data revealed a ‘proximity phenomena’ that appeared promising to better understand the emergence, in the evolving network, of mutual awareness (comprehend other network actors’ objectives and practices) and relationships that might encourage and facilitate integrated network-based practices. In the secondary analysis presented in this article, we ask: How do deliberate actions bring individual and organizational actors closer together? What conditions their ability to interact?

### Proximity as a new theoretical approach

Proximity theory [14,15] distinguishes multiple dimensions that play complementary roles in enabling integrated practices. It aligns with Valentijn’s Rainbow Model of Integrated Care [5,6], in which functional and normative integration at micro, meso and macro level foster clinical, professional, organizational and system integration. Proximity theory complements this view by helping explain how and why relationships take shape as operating mechanisms for integration. Proximity also facilitates understanding of how integration is supported in complex contexts such as cancer networks and how it is sustained as context changes over time. Proximity is interested in relationships and the role of perceived space in enabling coordination in innovative sectors [16]. The proximity literature generally reports five dimensions of proximity [17], with some adding a sixth dimension of technological proximity [14] – reflecting the growing importance of eHealth and the COVID-19 pandemic. Table 1 provides definitions of each dimension and illustrative examples of real-world network-based practices in cancer care.

**Table 1:** Dimensions of proximity and illustrative examples in cancer care.


DIMENSIONS OF PROXIMITY	ILLUSTRATIVE EXAMPLES IN CANCER CARE

Geographic proximity can be objective (metric distance) or subjective (perceived) [18]. It involves face-to-face or virtual interactions among permanently or temporarily (i.e. meetings) co-located actors that enable information exchange [19].	Acknowledging the impact of traveling time and cost on professional coordination and as a contributor to access inequalities for cancer patients in rural or remote areas Co-locating clinicians in a Comprehensive Cancer Center to advance integrated care, and considering the potential impact on inter-organizational transitions along the cancer continuum Considering (for both clinicians and patients) the spatial dilemmas involved in establishing hierarchies of services according to complexity of cancer type

Relational proximity involves trust and mutual respect between actors [20] that recognizes their interdependencies	Facilitating the quality and quantity of communication Supporting professional positions in the integrated network and commitment to the cancer care continuum Improving the feeling of individual attachment to the local and national cancer network

Cognitive proximity entails shared mental models of a situation [14] which is useful in interdisciplinary teamwork and transitions between cancer and primary care teams	Making sense of notions of “Cure” and “Care” beyond disciplinary knowledge Recognizing tensions between PLC experience and clinical expertise in patient-centered care Providing interdisciplinary training on the goals and processes of teamwork

Organizational proximity describes routines and processes that reduce the transaction costs of interactions within or between organizations [21]	Aligning organizational structures among different independent organizations (e.g. hospital, primary care, home care, non-profit community organizations) and coordinating interdependencies Establishing multiple levels of network governance (national, regional, local)

Institutional proximity touches on the roles, norms, culture and values of a field [14,17,21]	Facilitating top-down and bottom-up linkages between national cancer priorities and communities of practice Supporting a consultative committee structure Shifting from vertical government “prescription” to forms of collaborative governance

Technological proximity refers to the tools that help actors interact and understand each other [21], and support knowledge exchange and interactions [14,18,21].	Transforming the perception of space using virtual communication technologies Sharing knowledge during virtual tumor board and interdisciplinary team meetings to support treatment decision-making and shared goals. Using eHealth to improve patient–provider communication (symptom and toxicity assessment and management, optimising patient engagement)


**Figure 2:**
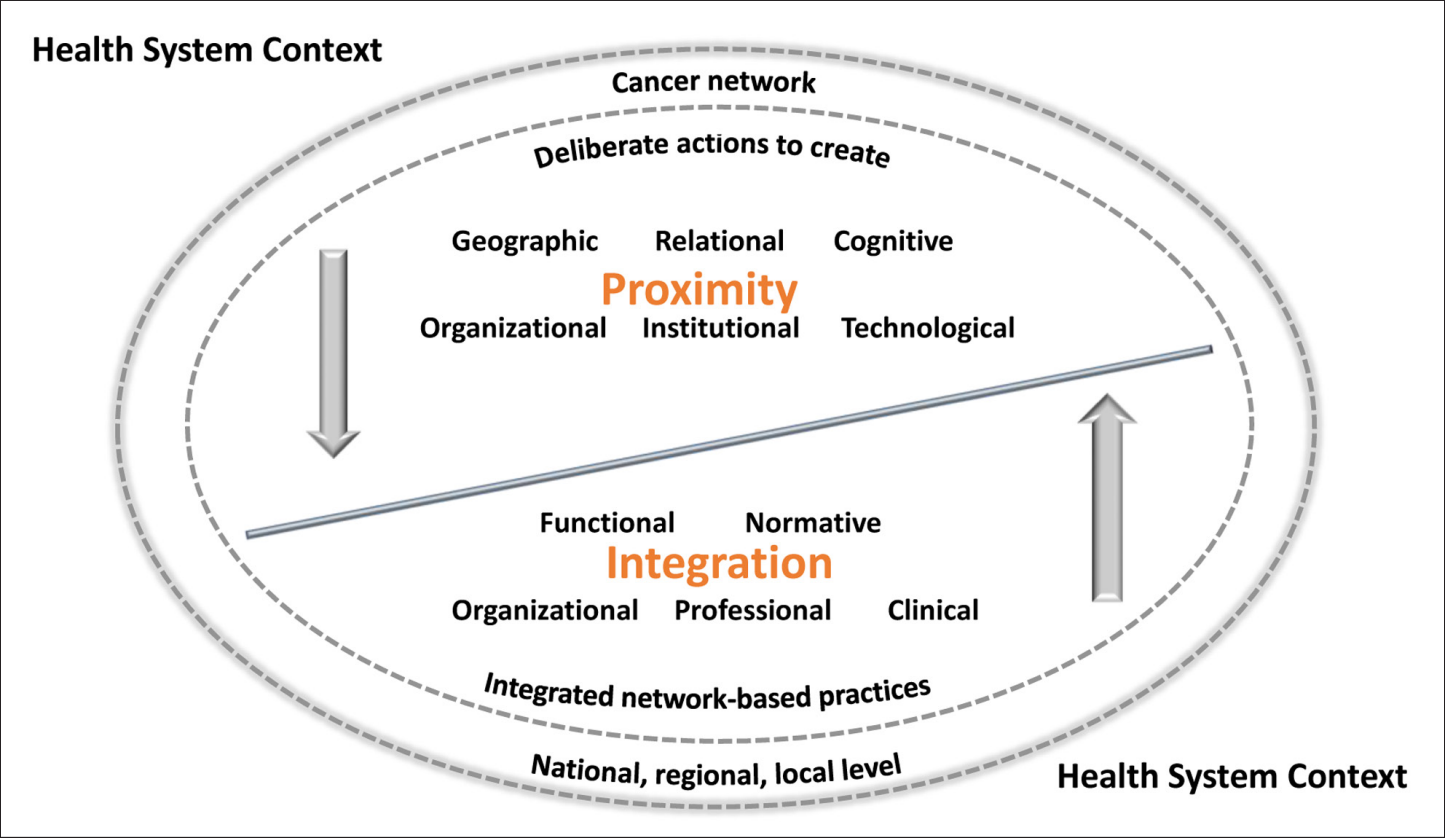
Dimensions of proximity as mechanisms underpinning integrated practices.

There is considerable debate among proximity scholars around the extent to which proximity dimensions exert a distinct influence [14,22]. For example, some authors consider that organizational proximity also encompasses cognitive, institutional, cultural and social dimensions of proximity [23]. Boschma [17] sees the dimensions as intrinsically interrelated and acting upon each other in various ways, by generating or supporting other dimensions, attenuating the effect of other dimensions, or compensating for weakness of other dimensions. For example, relational proximity may compensate when institutional proximity is weak. Although authors use somewhat different definitions for similar dimensions of proximity, and find overlap between these dimensions in empirical analysis [14], general agreement exists that coordination dysfunctions can be explained by the various dimensions of proximity [17]. Figure 2 depicts the complementarity between dimensions of proximity and dimensions of integration that appear conceptually as plausible underlying mechanisms associated with integrated practices in cancer networks. In a highly contextualized way, various dimensions of proximity may activate functional and normative mechanisms to ensure connectivity between organizational, professional and clinical dimensions of integrated care. As shown in Figure 2, progressive increases in integration then exert a positive influence on dimensions of proximity, creating a virtuous cycle.

Proximity theory appears as a useful guide to identifying deliberate actions in a network that interact and accumulate to nurture sustainable integrated practices. Network models in health care seek to address gaps arising from fragmentation by generating shared mental models and coordinated practices to assure a smooth continuum of care [24]. Actions are dependent on context at multiple levels (national, regional, local) in the cancer network, which is itself embedded in the larger health system.

## Methods

### Study design

The secondary analysis [25] presented here is based on qualitative data previously collected by the researchers (DT and NT as Co-PI) in a study [7] centred on core concepts of collaborative governance and network integration. The primary study used a nested multi-case study design, accounting for the “network-of-networks” developed in Quebec; this design is especially suited to in-depth exploration of complex phenomena that are highly context dependent [26]. Our secondary qualitative data analysis [27] transcends the focus on particular forms of integrated network-based practices to examine an underlying emergent aspect – proximity phenomena – using a new theoretical approach. The design for the secondary analysis is Interpretive Description (ID) informed [28] by the framework depicted in Figure 2.

### Setting and participants

The secondary analysis focused on qualitative data collected from three regional cancer care delivery networks embedded in the QCN, selected based on variations in their characteristics (geography, population, mandate, number of network organizations, cancer services provided). This choice was not made in order to compare regional networks, but rather to include a range of experiences and perspectives [29] regarding relationships and interactions across the network-of-networks. A convenience sample was built to include comprehensive representations from key informants involved in regional and/or national network governance committees where network-based practices are deliberated. Informants also experience these practices at various levels in the field, and their perceptions point to the creation of proximities that influence relationships and interactions within and between levels of the cancer network. Inclusion criteria were to have knowledge and experience of deliberate actions undertaken to integrate cancer care and to have lived experience of committee meetings.

### Data collection

Qualitative datasets from the primary study (collected between November 2018 and February 2020) relied mainly on individual semi-directed interviews with 36 key informants (see Table 2), including three who have a dual function as clinician and policy-maker. Ethics approval was received from the Research Ethics Committee (Project number: MP-04-2019-316) and informed consent forms were signed by all participants.

**Table 2:** Characteristics of participating sites and key informants.


CHARACTERISTIC	SITE 1	SITE 2	SITE 3

Geography	Rural+Semi-urban+Urban	Urban	Rural+Semi-Urban

Territory (Km2)	1,391	88	15,074

Population on territory	420,000^a^	446,800^a^	424,856^b^

Mandate	Community	Academic	Community

Number of network organizations	19 (2 CH, 7 CLSC, 10 GMF/UMF)	11 (3 CH, 6 CLSC, 12 GMF/UMF)	71 (5 CH, 26 CLSC, 39 GMF/UMF)

Cancer services provided	Radiotherapy: Yes Integrated cancer centre: Yes	Radiotherapy: No Integrated cancer centre: No	Radiotherapy: Yes^c^ Integrated cancer centre: Yes

Key informants/sites			

Clinicians	6^d^	5^d^	5

Managers	3	3	3^d^

PLC representatives	1	1	1

Non-profit Org. leaders	0	0	2

Total/sites	11	9	11

Key informants/QCN	7		


LEGEND: ^a^: Population in 2020; ^b^: Population in 2018; ^c^: Integrated cancer centre and radiotherapy facility opened in January 2019; ^d^: Key informants involved at two levels. CH: Centre hospitalier (Hospital); CLSC: Centre local de services communautaires (Local community service centre); GMF: Groupe de médecine familiale (Family medicine group); UMF: Unité de médecine familiale universitaire (Academic family medicine unit); QCN: Quebec cancer network.

Interview guides and data collection grids were based on the collaborative governance framework, focussing on coordination arrangements in the cancer network [7]. Collaborative governance [30] describes interacting components that optimize capacities held by various actors and coordinates them towards a common goal: (1) principled engagement (process of discovery, definition, deliberation, collaboration); (2) shared motivation (increases trust as a result of collaboration and fosters further principled engagement; (3) capacity for joint action (integrated network-based practices along the cancer trajectory). Components of collaborative governance aim to bring actors closer together to address fragmentation in the patient experience of care. The interview guide therefore generated responses that were well-suited to making new connections within the data.

Interviews for the primary study (average 60 min) were conducted by co-authors familiar with the cancer network (DT, NT, JC), audio-recorded and transcribed. Data also included documents published between 1998 and 2020 (e.g. strategic and national action plans, local and government reports, non-participant meeting observation (n = 28, total hours) and informal conversations by the first author to gain a sense of actors’ attitudes and perspectives on the complexities of integrated practices [28]. Qualitative datasets were integrated in a single database and handled using QDA Miner 5^TM^ software [31].

### Data analysis

The data analysis process involved abductive reasoning [32]. It offers the opportunity to dive into the actions undertaken to promote integrated practices and empirically explore the contribution of proximity theory. Qualitative analysis proceeded in sequential coding cycles [28,33]. Initial analysis (SU, JC) involved structural coding based on the interview guide. Raw data were then grouped into categories, and it was during this descriptive cycle that the dimensions of proximity assumed significance within efforts towards engagement, shared motivation and joint action. We (DT, NT, SU, JC) then undertook an interpretative cycle to “re-construct” elements found in the raw data [34] and explore proximity dimensions that emerge through collaborative governance efforts and serve as mechanisms to enable integrated network-based practices. The re-construction was performed using matrices (Excel) to identify relevant relationships and interactions, along with supporting interview extracts [33]. These matrices were analysed independently by all four co-authors, and discussed to reach consensus. Appendix 1 supports the transparency of the analysis by presenting quotes illustrative of participant perceptions of the actions that generated different dimensions of proximity.

## Results

The analysis reveals actions that served to create or enhance dimensions of proximity among network actors and their influence on features of integrated practice in cancer care. In this section, we present main findings regarding the six dimensions of proximity, and a selection of illustrative quotes from study participants.

### Geographic proximity

A number of actions created geographic proximity to align activities and promote functional and normative integration of practices in the network. At both national and local level, opportunities were created to bring professionals from different disciplines and different regions into the same physical or virtual space on a regular, but temporary basis. National Coordinating Committee meetings held in person or by videoconference gave network members an opportunity to learn about QCN objectives and their colleagues’ activities and challenges to integrate practices. Geographic proximity fostered principled engagement, providing a basis for further endeavours to mobilize and coordinate intra- and inter-disciplinary teamwork and pave the way for relational and cognitive proximity.

*At local coordinating committee meetings, we each provide a status report from our own areas. Everyone at the meeting is then able to bring back to their milieu an account of what came out at the meeting, the broad orientations, so they can all work in the same direction. (Healthcare professional, Regional cancer network)*

Communities of practice, supported by national network leaders, brought together professionals from different local regions, enabling them to share best practices in a given domain and spread them across the network. They also helped some hospital-based professionals develop trust to transition patients to colleagues in primary care. A community of practice for PLC enhanced their confidence in contributing to shaping network services to respond to patient needs.

Opportunities to enter the space of other actors, such as through site visits, were another means used to create geographic proximity, and appeared especially important to recognizing the capacities of colleagues and building the trust required to coordinate the distribution of services and referrals in the network. Geographic proximity appears as a precursor to relational and cognitive proximity and supports clinical, professional and organizational integration.

*[Professionals] had to go see for themselves what was available in the hospital where we were referring patients, to see that the quality of care would be just as good. It was a valuable learning experience. (Physician, Regional cancer network)*

Project planning workshops and work to design integrated networks for specific tumour sites created geographic proximity that was especially propitious to cognitive and relational proximity (see below) as actors came to appreciate each other’s contributions to addressing whole person needs. These findings support the idea that temporary geographic proximity can enhance mutual awareness, knowledge transfer and contribute to integrated network-based practices.

### Relational proximity

Relational proximity is essential to trust and affinity between actors that motivates collaboration and supports continuity of care. A number of deliberate actions taken to pursue collaborative governance appeared to generate relational proximity between national and local leadership and between local leadership and providers.

The National Coordinating Committee served as a key vehicle to communicate network objectives to administrative and clinical directors in local regions. While Committee meetings helped create cognitive proximity as leaders in each region heard the same messages and expectations, they were less effective at generating relational proximity and trust between regional and national levels. Local actors had little opportunity to express and discuss their concerns. There was a perception that the agenda was pre-set at the national level and minimized regional specificities or local challenges. Some local actors expressed actual mistrust of national motives, especially around performance indicators, which they perceived as means of control.

Local leadership was responsible for implementing national network directives within their region. In the mega structures of the IHSSC, leadership appeared especially important to creating the relational proximity needed to enlist and support operational actors in implementing top-down network prescriptions. Respondents in local regions suggested that relational proximity was created through accessible and shared leadership styles, opportunities for discussion, and a non-judgmental approach to problems. This openness was seen to characterize local multidisciplinary coordinating committee meetings, enabling participants to bring problems to the table and creating an atmosphere of mutual trust, reliance and responsiveness. This relational proximity facilitated clinical and professional integration at the local level.

*Everyone is there [at local committee meetings], we talk about what’s really going on… even when there’s a problem, people put it on the table. We’re not about appearances. (Physician, Regional cancer network)*

Actors at local level who also had roles at national network level served a linking function that helped overcome barriers to trust between levels.

Relationships between local actors on different sites – even from the same profession – had to be actively cultivated to build trust for referrals, especially between specialized cancer teams and primary care providers.

*The teams didn’t know each other. There’s a lack of trust… physiotherapists often keep patients longer in hospital because they think: We can’t send them to primary care, we don’t know what they do on a home visit. (Director of professional services, Regional cancer network)*

The communities of practice, where professionals could identify and solve problems together, supported relational proximity. Emphasis on patient-centred care in the network (see cognitive proximity below) enhanced relational proximity by providing a focus around which actors, even with divergent interests, could build the collaborative relationships needed for integrated practice.

### Cognitive proximity

A number of actions were taken in the network to create cognitive proximity. The National Cancer Plan was promoted consistently by a highly credible and stable leadership team. The Plan’s foundation in evidence and person-centred care heightened its impact in aligning actor perspectives and creating cognitive proximity. Ministry leadership was generally appreciated as it provided clear vision and direction. However, the obligations that flowed down from the Ministry sometimes clashed with local realities or priorities, making coordination more difficult. The transmission of information from local to national levels, and the national level’s ability to address local concerns and ambitions, was tenuous. The lack of cognitive proximity between levels risked jeopardizing organizational and systemic integration as local actors faced conflicting imperatives and their local improvement efforts went unrecognized by national network leaders. Within professional groups, the geographic proximity produced in communities of practice enhanced cognitive proximity as best practices were developed, adopted and spread across local networks.

At local level, interdisciplinary teams created cognitive proximity by bringing actors together to exchange on clinical issues, enabling them to reframe their conception of cancer care to incorporate the contributions of others. Committees allowed actors to become aware of challenges experienced by other team members and see where collaboration was needed to resolve them.

*The national and local committees enable knowledge sharing… we’ve never been so aware of what’s going on in other regions. (Clinical manager, Regional cancer network)*

Emphasis on patient-centred care contributed to cognitive proximity by providing a common objective around which multiple actors could coordinate and converge.

*The vision of patient-centred care has a lot of power and inspires confidence in the decisions we make. The patient experience forces us to develop concerted action. (Director of professional services, Regional cancer network)*

The effect on cognitive proximity was enhanced when PLC input challenged provider assumptions about what mattered to patients. A PLC community of practice enabled the PLC who participated on local committees to share their experience and expertise and shape priority issues that could then be communicated consistently back to each local region. These deliberate actions within the network to create cognitive proximity appeared to support clinical and professional integration.

### Organizational proximity

Organizational proximity was created at two levels in the network: the National Cancer Network that communicated a vision and established norms for operations; and action plans at local level.

Visionary leadership was recognized as driving the organization of a national “network of networks” and imparted a sense of coherence to the rearrangement of services. The National Cancer Plan, strongly associated with a respected leader in cancer, was powerful in orienting actions; the Plan’s influence within the Ministry helped local leaders push cancer care as a priority in their organizations.

*Dr (Name) succeeded in maintaining, at local level, a cancer care governance that doesn’t exist in other specialties. There is no dedicated governance of cardiology or other chronic disease in the hospitals, and that’s what makes all the difference. (Ministry planning actor, National cancer network)*

Expectations from the national level created proximity among organizations that increasingly functioned in similar ways. Leadership at national level encouraged harmonization by issuing comparative portraits of local establishments based on performance indicators. The Cancer Plan promoted an organization of services that required local networks to concentrate specialized cancer services and ensure transition to primary care for survivorship. Service agreements within and between local networks were beginning to enable a new distribution of roles and responsibilities and increase organizational proximity across establishments.

Many respondents considered that the creation of the IHSSCs in 2015 contributed to organizational proximity in local networks. The new structures brought multiple services under common leadership and governance, facilitating both horizontal and vertical integration: more efficient arrangement of specialized cancer care in a region, and a push for referrals between providers across the cancer continuum. PLC participation in decision-making helped overcome disputes over where services should be provided, supporting organizational proximity. Common governance of facilities within each IHSSC created organizational proximity by encouraging practice harmonization.

*Before the IHSSC, the three establishments were very divided. Now we work under a single executive team and go to the same meetings. We hear the same messages, so there’s more cohesion, more exchange. (Oncology lead, Regional cancer network)*

Sometimes, however, this risked a downward levelling of services when resources were uneven across facilities.

Efforts to create organizational proximity were less apparent between levels of care and were impeded by a poor understanding, among specialized cancer teams, of the primary care sector. Neither were communication problems satisfactorily addressed to help in this regard, especially during transition to family physicians and non-profit community services, where information flow was insecure, relational proximity non-existent and communication pathways weak.

### Institutional proximity

The strategic position of the Cancer Directorate within the Ministry enhanced institutional proximity among cancer care providers at system and organization level. Efforts were made to support shared norms and values, joint action and optimize scope of professional practices within the network, notably through communities of practice and interdisciplinary teamwork.

*One of the first things we did (in the community of practice) was to describe the role of the pharmacist in oncology. Surprisingly, no one had ever done so, because it’s not a recognized title in the hospital structure. (Ministry planning actor, National cancer network)*

The network provided some cancer specialists opportunities to strengthen collaboration and knowledge exchange amongst each other across IHSSCs. For others, it raised controversies around service hierarchies and formalized referral patterns. While professionals benefitted as their organizations were designated and resourced to improve integrated network-based practices, the payment model for physicians (fee-for-service), their clinical autonomy and sole responsibility for patient admission and discharge from cancer care maintained a distance between physicians and policy orientations.

Efforts were not entirely effective at safeguarding the cancer network as an institution at the policy level. For example, in 2019, the Ministry downgraded cancer care from a Directorate to a Program. At the care provision level, the objective of extending the role of primary care confronted institutional barriers related to practice context that superseded professional institutions.

*As family physicians, we transfer responsibility to cancer specialists, often without hearing back from them. (Primary care physician, Regional cancer network)*

*It seems like family physicians don’t feel competent… to keep following the patient. (Oncology lead, Regional cancer network)*

A disconnect between national discourse and actions to pursue this objective prevented creation of geographic, relational or technological proximities that could have provided a starting point for developing institutional proximity.

### Technological proximity

Tools such as the national cancer registry, shared medical records and shared indicators helped create technological proximity, encouraging actors to adopt similar language, focus on similar preoccupations and, to some extent, coordinate care between providers. While a very basic shared record was available for scans and lab results, respondents expressed a need for more detailed communication between providers to assure safe transitions. Reliance on electronic communications and central intake systems for referrals precluded relationship building between different specialists, as well as between hospital and community-based providers.

*(The radiologist) says: refer the patient through the central intake system with the appropriate priority. But we would really benefit from actually speaking to each other rather than communicating via a form. It gives us the impression they don’t really have time to talk to us. (Healthcare professional, Regional cancer network)*

The cancer registry provided a basis for concerted action in service design and planning, bringing a level of objectivity to resource allocation decisions. Closer links between managers and registry personnel, established in local committees, helped ensure these data were useful and used. The national level used monthly video conferencing to share best practices at local level that improved performance on indicators, helping harmonize practices across the network and contributing to system integration.

## Discussion

### Achieving integrated network-based practices by creating proximity

To our knowledge, this study is the first to associate dimensions of proximity with deliberate actions within a cancer network. Findings provide insight and guidance to practitioners seeking to promote integrated practices. They reveal how efforts to overcome the challenge of fragmentation in cancer care may be supported by the proximity framework. Coordination problems must be addressed by the various dimensions of proximity [17]. Interactions at different levels in the network enhance dimensions of proximity, though they are less successful at creating institutional proximity, potentially due to professional culture and specialization [35] which others have associated with inertia in health systems [36].

### Synergy between actions: an essential ingredient to enhance proximity

Findings suggest that the influence of network actions depends on their coherence with each other [37]. As one example, reliance on PLC participation [38] as a lever to create relational and cognitive proximity rested on several actions operating in synergy: 1) integration of PLC in national and local governance committees, 2) implementation of a community of practice bringing together PLC from across Quebec; and 3) the national framework for PLC partnership. Taken together, these deliberate actions reinforced normative integration by enabling the emergence and spread of a common view of network objectives, and shared values around person-centred care.

A similar synergy is seen between actions to support communities of practice [39] that enhance institutional proximity within a given profession, and interdisciplinary committees at local level that create the cognitive and relational proximity essential to clinical and professional integration. In integrated cancer networks, distance between uni-professional communities of practice can compromise knowledge sharing and collaborative interprofessional practices [40]. Our study shows that the cancer committees at local level offer a “sustained platform of joint activity” [40]; they encourage teamwork and shared learning among professionals who are simultaneously engaged in communities of practice to enhance their own professional practice. This pattern reflects the balance of proximity described by Knoben and Oerlemans [14]. “Units” pursue new knowledge in communities of practice and can then introduce this knowledge in local multidisciplinary committees to see how it fits with other contributions. This cognitive proximity for multi-level problem solving promotes recognition of interdependencies and the value of interdisciplinary teamwork in cancer care [41]. Scholars looking at proximity [14], like those looking at networks [42] are concerned that learning and innovation not be lost through an overabundance of cohesiveness. This is especially important in networks with strong central leadership – in our case the national cancer directorate – where integration risks veering toward rationalization of resources and jeopardizing integration of providers [43].

### Importance of managing tensions related to network leadership

Strong and respected leadership is a lever to enhance proximity and overcome health system inertia [44] while also assuring continuity in the effort to integrate practices. Network leadership was seen as a beacon, keeping objectives alive though the turbulent context of health system reforms, providing consistent vision, stewardship of professional communities of practice, facilitation of committees and promotion of person-centred care that enhanced multiple dimensions of proximity.

However, emphasis on institutional and organizational proximity in the QCN, imposed through prescribed elements (e.g. hierarchical organization, standardization of indicators, integration of primary care providers), was seen as threatening to local networks that each faced distinct challenges. Given the tendency for trust between cancer care actors to erode under the weight of rules and norms induced by institutional proximity [45], deliberate actions (e.g. to support relational and cognitive proximity) may support professional and clinical integration and reduce fragmented care [4]. These results support that institutional proximity created through hierarchical mechanisms of governance based on bureaucratic structures and rigid rules compromises both horizontal and vertical integration [6]

### The complexity of developing and sustaining integration

Implementing network-based integration is complex, requiring that multiple dimensions of proximity be enhanced simultaneously. Technological proximity, for example, can be created through common indicators and shared medical records. However, such tools must be complemented by the creation of other dimensions of proximity, notably relational and cognitive proximity, to generate integration at clinical level; direct communication is needed to coordinate a patient’s care and be confident in referrals.

Research also suggests that while the cancer domain is an incubator of technological and therapeutic innovations, it is more resistant to institutional transformations that push the type of standardization of practices and processes [46] suggested by institutional proximity.

Finally, the study reveals a lack of proximity in several key areas that helps identify gaps in network action that may account for some of the shortfalls in integration. Referrals between professionals working in hospital and community settings remain problematic and participants describe issues with communication and trust that impede network-based practices. However, few actions are taken to create proximity, notably between specialist and family physicians, who figure as “missing actors” [47] in the integration project. Nominal mention in the Cancer Plan cannot create cognitive proximity without being backed up by the deliberate creation of geographic proximity within which other dimensions of proximity can develop. Neither is technological proximity created to palliate the gap, given that detailed medical records are not shared with community-based providers and automated referral systems limit the occurrence of detailed conversations between parties. These gaps leave little opportunity to develop relational proximity that can help develop the trust needed to overcome organizational and geographic divides.

Findings suggest that proximity offers a potentially actionable framework to support network efforts toward integration. They support the view that geographic proximity can serve to support cognitive, relational and organizational proximity, which in turn appear to support functional and normative dimension of integration in Valentijn’s model [6]. Results also shed light on how materiality influences the creation of proximity. There is increasing attention in the proximity literature to “virtual” proximity [22] as the digital age and now covid-19 make in-person meetings less necessary, desirable or safe.

However, technological proximity is not yet optimal and current information technology systems constrain the integration of practices along the cancer continuum [48]. Our results show that cognitive and relational proximity has less opportunity to emerge in virtual meetings [49], particularly when these are designed to impart rather than exchange information.

### Strengths and limitations

Supplementary analysis is recognized as an effective approach to maximize the use of original qualitative datasets to answer new empirical or theoretical questions relevant to the phenomenon originally studied [27]. In this case, it offered an opportunity to scrutinize how we might understand conditions that contribute to integrated practices without imposing on additional burden on study participants. We provide clarification in the methodology section on the boundaries between the primary study and the supplementary analysis [25], and refer readers to the primary study protocol [7] for further information. We recognize that this strategy may skew the emphasis toward proximity dimensions related to governance and organizational practices of integrated care. The generation of proximity dimensions important to clinical and professional integration may require the integration of theories of professionalism and leadership. However, our results reveal that geographic proximity (physical or virtual) drives relational and cognitive proximity, which appear to be associated with certain dimensions of integration – notably functional, normative and system dimensions of integration –, with less influence on professional and clinical integration. These findings should be explored in further primary study.

Interpretive Description as a qualitative research approach enables us to explore proximity through the actions and perceptions of actors at multiple levels in a cancer network. The prolonged commitment of the authors (DT, NT, JC) in fieldwork allows sustained observations and better control of biases that may result from premature conclusions [50]. The present small interpretive description study was not intended to achieve saturation. However, our results may be well received in the network context even without the benefit of data or theoretical saturation [28]. Data triangulation from multiple sources and co-analysis by research team members provide complementary perspectives for richer and more complete results [33] and help mitigate limitations arising from the convenience sampling and the sample size. The context of system modernization seen in Quebec will be familiar to other jurisdictions that have structured cancer services in network configurations. However, knowledge users should be cautious about the transferability of findings as with all contextualized qualitative studies in natural settings [51].

## Conclusion

This article is a first attempt to empirically mobilize the theoretical approach of proximity to understand actions that are more likely to generate integration in specialized healthcare networks. Dimensions of proximity, as a heuristic, can help researchers and network decision-makers plan, guide and evaluate actions to induce and sustain integrated practices.

Considering this supplementary analysis and our exploratory focus, results need to be tested in research on the creation of proximity in primary studies to more directly link actions to generate proximity with integrated service delivery. This may help move research from an exploratory into a more explanatory phase.

## Additional Files

The additional files for this article can be found as follows:

10.5334/ijic.6434.s1 Appendix 1. Table 1: Illustrative quotes from the analysis that support the transparency of the analysis.

10.5334/ijic.6434.s2 Appendix 2. COREQ checklist.
