# Supplementary material for: Dimensions of Proximity: An Actionable Framework to Better Understand Integrated Practices in Cancer Networks
Source: Int J Integr Care. 2022 Aug 16;22(3):9. doi: 10.5334/ijic.6434 (PMC9389948; doi:10.5334/ijic.6434)
Supplement: Appendix 1. — Table 1: Illustrative quotes from the analysis that support the transparency of the analysis. [file ijic-22-3-6434-s1.pdf]

APPENDIX 1:

Our descriptive interpretive study[1] integrates the perspectives of multiple actors in the Quebec Cancer Network. This Appendix presents samples of perspectives that lead us, in analysis, to identify actions generative of different dimensions of proximity. Acknowledging that not every theme (proximity dimensions in our study) requires multiple quotes[2,3], especially illustrative quotes help communicate the logic behind researcher interpretations and increase transparency.

Table 1. Illustrative quotes from the analysis that support the credibility of the analysis

Action taken	Illustrative quotes
**Actions that generate geographic proximity**	
Establishment of coordinating committees to bring actors together	One of the first venues was the National Committee of clinical managers. At Ministry level, coordinating committees and certain committees by tumour site were established. These steps put the basic networking structure in place. (Ministry planning actor, National cancer network)<br><br>At local coordinating committee meetings, we each provide a status report from our own areas. Everyone at the meeting is then able to bring back to their milieu an account of what came out at the meeting, the broad orientations, so they can all work in the same direction. (Healthcare professional, Regional cancer network)
National support for professional communities of practice	For pharmacists, it's clear that the Cancer Directorate support to sustain this community of practice is essential. The network expanded the community of practice from the large hospital centres into more distant regions. Today ... it spans the entire province. (Ministry planning actor, National cancer network)<br><br>[the community of practice in psychosocial oncology] enables us to get to know each other. As a hospital-based social worker, the opportunity to meet with social workers from primary care means I know what's available for patients if I refer them to care in the community. I know what services they offer. I know their wait times. I know their challenges. I trust my front-line partner. (Healthcare professional, Regional cancer network)
Opportunities for actors to enter the space of other actors	[Professionals] had to go see for themselves what was available in the hospital where we were referring patients, to see that the quality of care would be just as good. It was a valuable learning experience. (Physician, Regional cancer network)<br><br>Implementing a network requires accompaniment so we [National network leaders] are present. We help them make contact, but once that first link is established, we step back a little. (Cancer Directorate actor, National cancer network)
Participation in development of particular projects	...there were many meetings to get [the new cancer centre] going so people know each other well, they talk to each other. (Oncologist-manager, Regional cancer network)

Actions that generate relational proximity	
Meetings between national leaders and directors of local regions	We want to enact the Plan, but we also want to ensure that we meet the objectives in a way that is locally appropriate. (Physician, Regional cancer network)<br><br>At national level, they claim to want to be close to the local regions, but that's not what happens. They look to us for information to justify their own positioning. (Director of professional services, Regional cancer network)
Collaborative local leadership and a problem-solving approach	Before coming here, I had never experienced this kind of participation. We're often asked: What do you think? Does this make sense to you? Everyone participates, everyone collaborates, everyone feels involved. (Healthcare professional, Regional cancer network)<br><br>Everyone is there [at local committee meetings], we talk about what's really going on... even when there's a problem, people put it on the table. We're not about appearances. (Physician, Regional cancer network)<br><br>Here, I think we benefit from a very direct culture. People all know each other, so things get done, calls are returned very quickly. (Senior leader, Regional cancer network)
Linking actors to carry issues up, down or across	In the past few years we felt it less at the policy level...then Ms (Name 3) came to our meeting and we could see that the problems we were describing were really understood. I felt there was a real willingness to take charge and get things moving. (Healthcare professional, Regional cancer network)<br><br>Professionals are involved in local committees and involved in various professional communities of practice, so there's cross involvement at many levels. (Director of professional services, Regional cancer network)
Site visits to build relationships between hospital and community actors	The teams didn't know each other. There's a lack of trust... physiotherapists often keep patients longer in hospital because they think: We can't send them to primary care, we don't know what they do on a home visit. (Director of professional services, Regional cancer network)<br><br>Our psychosocial team went to present to all the pivot nurses in oncology, but they are so absorbed in daily work that they quickly forget what's available in primary care. (Director of front-line services, Regional cancer network)

Actions that generate cognitive proximity	
Consistent promotion of National Cancer Plan	The National Cancer Plan... led to sharing knowledge around care and approaches. Our teams speak the same language and identify the same priorities (Clinical manager, Regional cancer network)<br><br>Personally, I don't think it's a bad thing that it [the Cancer Plan] comes from the top. There were analyses, evidence, so I'm very comfortable taking what is prescribed and bringing it to my team. I trust what's being prescribed. (Director of professional services, Regional cancer network)<br><br>*BUT... dissonance between Plan and local realities*<br><br>When our needs correspond to a [national priority] and our reality adheres to the prescribed model, they listen, we get support and it can be quite effective. On the other hand, there isn't much room for difference because we're constantly trying to harmonize. But standardization has limits. (Clinical manager, Regional cancer network)
Professional communities of practice and interdisciplinary teams	The psychosocial community of practice works through collaboration, partnership. We're aware what's being done and how it's being done. We identify gaps and successes. (Healthcare professional, Regional cancer network)<br><br>The national and local committees enable knowledge sharing... we've never been so aware of what's going on in other regions. (Clinical manager, Regional cancer network)<br><br>Local committees involve working in small mixed groups... I might find myself with a specialist in oncology, a nurse, a patient and the guy from information technology. It makes the meetings very interesting and helps the knowledge and concerns of each participant get attention. (Primary care physician, Regional cancer network)
Participation of PLC in committees and projects	The vision of patient-centred care has a lot of power and inspires confidence in the decisions we make. The patient experience forces us to develop concerted action. (Director of professional services, Regional cancer network)<br><br>What PLC (on the local committee) were saying was listened to because it resonated with what was being said in the National PLC committee. (Clinical manager, Regional cancer network)<br><br>When IHSSC were competing or disagreed on something, bringing the conversation back to 'Yes, but what does the patient want?' would often calm things down and we could come to an agreement. (Cancer Directorate actor, National cancer network)

Actions that generate organizational proximity	
Visionary leadership at national level to carry the National cancer program	The cancer program provides direction... actions that are concerted, structured, with clear orientations; I find that very helpful for an organization. (Nursing director, Regional cancer network)<br><br>Dr (Name) succeeded in maintaining, at local level, a cancer care governance that doesn't exist in other specialties. There is no dedicated governance of cardiology or other chronic disease in the hospitals, and that's what makes all the difference. (Ministry planning actor, National cancer network)<br><br>The vision (Cancer Plan) greatly benefits our organization, to move ahead on projects that had been dragging for years. (Oncologist-manager, Regional cancer network)
National prescription of common standards of practice to be adopted by local organizations	The Ministry imposed rules, and while we may not always like it, they do seem to have reframed things a bit. In the beginning, everyone felt like they were being watched and obviously didn't much like that, but after a while, when you think that they're for the good of the patient... they provide a framework ... then we can manage the exceptions, the different realities, but at least the standards are out there. (Healthcare professional, Regional cancer network)<br><br>Our local committee makes sure we apply what comes down from the Cancer Directorate... such as new guidelines for pivot nurses. (Oncology lead, Regional cancer network)<br><br>It was really the national level that helped us mobilize the oncologists in tumour boards. Being a provincial network helped... and now you really sense that it's important. We're no longer in obligation and prescription: we're really working in the interest of the patient. (Senior leader, Regional cancer network)
Formalizing roles and responsibilities across local network actors	We look at how each can contribute to support the other. For example, with lung cancer, thoracic surgery can only be done in a designated centre. We developed formal transition protocols in collaboration with the centre to benefit from their expertise. Same for radiation oncology, so we try to find solutions where they can provide us the service more formally (Oncology lead, Regional cancer network)<br><br>We formalized a structure (hierarchical network) with designated referral centres, affiliated centres, partner centres, defined each one's role and traced where accountability begins. (Cancer Directorate actor, National cancer network)
Common leadership and governance of sites within a local network under the IHSSC.	The IHSSC was a revolution, encouraging hospital staff to look outside their speciality and integrate primary care providers. The advantage of the IHSSC is that everyone is sitting together and the "We" becomes "we the IHSSC". (Physician, Regional cancer network)<br><br>Before the IHSSC, the three establishments were very divided. Now we work under a single executive team and go to the same meetings. We hear the same messages, so there's more cohesion, more exchange. (Oncology lead, Regional cancer network)<br><br>When connections were created between the four hospitals, which didn't exist beforehand, practices all started to harmonize. (Senior leader, Regional cancer network)
Expanding role of primary care	It (the role of primary care providers) is still quite abstract. We understand the vision of a cancer continuum but it's not

providers	yet concrete in terms of integrated professional or clinical practices. (Director of front-line services, Regional cancer network)<br><br>At the hospital, the cancer team will conduct comprehensive assessments, but when the patients transition back in primary care, they repeat the assessment. The Deputy Minister sent a clear message: I don't want to see any more duplicate assessments anywhere in Québec. (Director of professional services, Regional cancer network)

Actions that generate institutional proximity	
Status of the Cancer Directorate	It (the Cancer Directorate) is one of the more structured directorates within the Ministry. We know where it's heading, and where we're heading: we have clear goals and feel it's always in the interest of the user. (Oncology lead, Regional cancer network)<br><br>The shift from a Cancer Directorate to simple "Program" removed our issues from the table and reduced our levers to ensure the evolution of the Cancer Network. We have to find new ways to make sure our priorities are considered in the Ministry. (Ministry planning actor, National cancer network)
Distinct professional roles in cancer care are promoted...<br><br>... but not sufficiently protected	One of the first things we did (in the community of practice) was to describe the role of the pharmacist in oncology. Surprisingly, no one had ever done so, because it's not a recognized title in the hospital structure. (Ministry planning actor, National cancer network)<br><br>As a pivot nurse in oncology, you identify with that role, you're called a pivot nurse and you're a member of a solid community of practice. But on the human resources list, you're a clinical nurse, and can be moved to departments outside cancer. (Nursing lead, National cancer network)
Formalization of referral patterns and cancer care trajectories around the patient	Having a trajectory sparked the interest and participation of physicians. Everyone got involved and now we're really thinking we need to design other trajectories. (Senior leader, Regional cancer network)<br><br>We (designated referral centres) are not here to steal your patients... often what makes things work or not is tied up with remuneration. (Senior leader, Regional cancer network)<br><br>*BUT... Lack of institutional proximity between specialist and primary care physicians*<br><br>As family physicians, we transfer responsibility to cancer specialists, often without hearing back from them. (Primary care physician, Regional cancer network)<br><br>It seems like family physicians don't feel don't feel competent... to keep following the patient. (Oncology lead, Regional cancer network)<br><br>I think it's more a matter of clarifying expectations of the specialist if they refer the patient to primary care: what type of problem do they expect to be dealt with versus what kinds of problems primary care feels should be solved by cancer teams. (Director of professional services, Regional cancer network)<br><br>Primary care gets minimal attention in the National cancer program, which really focuses on super-specialization. (Director of front line services, Regional cancer network

**Actions that generate technological proximity**	
Shared medical records and referral platforms	Informal discussions are more difficult now because we're no longer co-located. Referrals are now electronic. We lose important clinical information there. Basically, we used to talk and now it's on the computer. (Healthcare professional, Regional cancer network)<br><br>(The radiologist) says: refer the patient through the central intake system with the appropriate priority. But we would really benefit from actually speaking to each other rather than communicating via a form. It gives us the impression they don't really have time to talk to us. (Healthcare professional, Regional cancer network)<br><br>Often, we (pivot nurses) refer a patient to primary care through an electronic system. We rarely get any feedback and it's the patient who keeps us informed of primary care interventions. (Healthcare professional, Regional cancer network)
National cancer registry	Before, the registry people had never met the cancer care coordinator or the administrative manager. They never had requests about registry data. Now I meet with the registry people, I go look at inputs, we do prospective studies on the characteristics of our clientele. We integrated the data in our IHSSC and created a platform that allows us to follow the patients' trajectories. (Clinical manager, Regional cancer network)<br><br>There's nothing more powerful than data. It's more powerful than money... it's very, very powerful in professional sectors. (Senior leader, National cancer network)
Best practice protocols, indicators and performance audits	There are audits done for waiting times for investigation, surgery, chemo and radiation Sharing regularly, we're able to identify problems and take action. Now wait times are closer to standards. (Healthcare professional, Regional cancer network)<br><br>We don't have the same clientele but we all have to satisfy the same indicators. Many indicators are related to cost. That can lead to perverse effects on person-centred priorities. (Oncologist-manager, Regional cancer network)

1. Thorne SE, Bultz BD, Baile WF. Is there a cost to poor communication in cancer care?: A critical review of the literature. Psycho-Oncology. 2005 Oct;14(10):875-84. https://doi.org/10.1002/pon.947
2. Wu, YP, Thompson, D, Aroian, KJ, McQuaid, EL, Deatrick, JA. Commentary: Writing and evaluating qualitative research reports. J Pediatr Psychol. 2016;41(5):493-505.https://doi.org/10.1093/jpepsy/jsw032
3. Sandelowski, M. (1994). The use of quotes in qualitative research. Res Nurs Health, 17, 479–482. https://doi.org/10.1002/nur.4770170611
