# Supplementary material for: Dimensions of Proximity: An Actionable Framework to Better Understand Integrated Practices in Cancer Networks
Source: Int J Integr Care. 2022 Aug 16;22(3):9. doi: 10.5334/ijic.6434 (PMC9389948; doi:10.5334/ijic.6434)
Supplement: Appendix 2. — COREQ checklist. [file ijic-22-3-6434-s2.pdf]

Additional file 2: COREQ[1] checklist

Table 1
Consolidated criteria for reporting qualitative studies (COREQ): 32-items checklist

No	Item	Remarks	Page/Line no
**Domain 1: Research team and reflexivity**<br>(Where blank, details are not mentioned in the article)			
Personal Characteristics			
1.	Research team	DT, NT, (Co-PI) and JC conducted individual interviews with participants. DT, NT, JC and SU conducted both primary study and secondary qualitative data analysis	Page/9 Line 11
2.	Credentials	NT, DT and SU are PhDs; JC a master degree	
3.	Occupation	DT and NT are professors and academic researchers; SU and JC are research professionals	
4.	Gender	All members of the research team are women	
5.	Experience and training	DT, NT and SU have training in qualitative methods and extensive experience in health services and qualitative interview-based research; JC, psychologist, is a seasoned health services manager with experience is knowledge uptake and transfer	
Relationship with participants			
6.	Relationship established	No relationship established prior the study. Any conflict of interest to mention	
7.	Participant knowledge of interviewer	DT was known by reputation to some participants as a researcher very involved in the cancer network since 2002.<br>The objectives and reasons for the study were described in the introduction section of the interview. (Appendix 2)<br><br>Participants were recruited following their expression of interest to a regional collaborator. Details are available in the primary study protocol (Tremblay, 2019), which we refer to in Section 2.1	Appendix 2
8.	Interviewer characteristics	DT is a senior career research fellow from the national research funder (Fonds de recherhe en santé-Québec). She has specific training in cancer services research, an advanced nursing background, and expertise in cancer network-based practices. NT has extensive experience in interview-based research in health services. JC has experience in interview-based research and is familiar with the cancer network. Details are available in the primary study protocol, which we refer to in Section 2.1	Page 9 Line 11

**Domain 2: Study design**			
Theoretical framework			
9.	Methodological orientation and theory	The secondary analysis is based on qualitative data previously collected by the researchers. The primary study used a nested multi-case study design, accounting for the "network-of-networks" developed in Quebec. Our secondary qualitative data analysis transcends the focus on particular forms of integrated network-based practices to examine an underlying emergent aspect – proximity phenomena – using a new theoretical approach. The design for the secondary analysis is Interpretive Description (ID) informed by the framework developed by our team and depicted in Fig. 2.	Page 9 Line 10-13<br><br>Page10 Line 2-6
Participant selection			
10	Sampling	A convenience sample was built to include comprehensive representations from key informants involved in regional and/or national network governance committees where network-based practices are deliberated.<br>Inclusion criteria were to have knowledge and experience of deliberate actions undertaken to integrate cancer care and to have lived experience of committee meetings.<br>Table 2 describes Characteristics of participating sites and key informants	Page 10 Line 13-15 Line 18-20<br><br>Page 11 Line 1
11.	Method of approach	For recruitment at regional level, collaborators in each site played a role in participant recruitment by informing regional actors (clinicians, clinician-managers, cancer program directors, PLC representatives) of the study and describing reasons they might wish to participate. Information meetings about the study were organised and regional collaborators used a formal recruitment script. They provided the research team with the names and contact information of actors interested in participating. The research team then contacted potential participants directly. Actors at national level were approached directly by the research team. For further detail, we refer readers to the primary study protocol (Tremblay 2019)	N/A
12.	Sample size	A total of 36 participants including three who have a dual function as clinician and policy-maker	Page 11 Line 5-6
13.	Non-participation	-	N/A
Setting			
14.	Setting of data collection	Individual interviews were conducted in private offices reserved within the cancer clinic or in the offices of	N/A

		interview participants. This information is available in the primary study protocol (Tremblay et al, 2019	
15	Presence of non-participants	No one was present besides the researchers and participants	N/A
16.	Description of sample	Individual interviews were conducted with clinicians (16) managers (9) PLC representatives (3), not-for-profit organization leaders (2) and key informants at national (QCN)level (7). See Table 2	Page 11 Line 1
Data collection			
17.	Interview guide	Interview guides (Appendix 1) and data collection grids were based on the collaborative governance framework, focussing on coordination arrangements in the cancer network	Page 11 Line 9-10
18.	Repeat interviews	-	-N/A
19.	Audio/visual recording	Interviews were conducted by co-authors familiar with the cancer network (DT, NT, JC), audio-recorded and transcribed	Page 12 Line 10-11
20.	Field notes	-	-N/A
21.	Duration	Average 60 minutes	Page 12 Line 10
22.	Data saturation	The present small interpretive description study was not intended to achieve saturation. However, our results may be well perceived in the network-based practice context even without the benefit of data or theoretical saturation	Page 27 Line 16-18
23.	Transcripts returned	No return to participants required considering audio recording	N/A
**Domain 3: Analysis and findings**			
24.	Number of data coders	2 (SU + JC) in cycle 1; 4 (DT, NT, SU, JC) in cycle 2	Page 13 Line 3
25.	Description of the coding tree	Proximity emerged as a promising theme following the initial structural coding of data based on the collaborative governance framework that informed our interview guide. A coding tree for Interpretive Descriptive analysis was then created based on the proximity dimensions to “re-construct” elements found in the raw data.<br>The re-construction was performed using matrices (Excel) to identify relevant relationships and interactions, along with supporting interview extracts. These matrices were analysed independently by all four co-authors, and discussed to reach consensus	Page 13 Line 1-6
26.	Identification of themes	Interpretive descriptive coding, using abductive reasoning focused on how dimensions of proximity were created through network actions.	Page 12 Line 19
27.	Software	QDA Miner 5™ software was used to manage data. Excel was used to create summary data tables.	Page 12 Line 17
28.	Participants feedback	Not involed in supplementary qualitative data analysis	N/A

Reporting			
29.	Quotations presented	Quotations are presented, identified by the participant's role (clinician, clinical manager, PLC...) and national or regional level of participation in governance committees (Results section and Appendix 2)	Page 14-22<br><br>Appendix 2
30.	Data and findings consistent	Findings are consistent with the data presented. All data were analysed in light of our conceptual framework. Quotations most illustrative of the data categories were selected by consensus from the team members for inclusion in the article.	Page 14-22
31.	Clarity of major themes	Findings are presented according to the six main dimensions of proximity: (3.1). Geographic proximity; (3.2) Relational proximity; (3.3). Cognitive proximity; (3.4). Organizational proximity; (3.5). Institutional proximity; (3.6). Technological proximity	Page 14-22
32.	Clarity of minor themes	Along with major themes, the discussion also pursues minor themes such as the interaction between proximity-generating actions in the network context and areas where actions were not taken to generate proximity. The discussion focuses on major themes considering the original findings that emerged from the data and their significance in relation to existing literature on healthcare integration. The identification of limits to local participation in governance functions of resource allocation and monitoring appear as a secondary theme	Page 22-26
